# Setting the stage for communication skills training in Rwandan cancer care: a qualitative study of local priorities and key contextual factors

**DOI:** 10.1186/s12904-025-01879-z

**Published:** 2025-09-30

**Authors:** Pacifique Uwamahoro, Ignace Girukubonye, Jean Bosco Bigirimana, Cyprien Shyirambere, Katherine Van Loon, Rebecca L. Sudore, Justin J. Sanders, Vincent K. Cubaka, Rebecca J. DeBoer

**Affiliations:** 1 Francois Xavier Bagnoud, Kigali, Rwanda; 2 Cordaid-Rwanda, Kigali, Rwanda; 3 Partners in Health/Inshuti Mu Buzima, Kigali, Rwanda; 4 University of California, San Francisco, San Francisco, CA USA; 5 McGill University, Montreal, Canada; 6 University of Global Health Equity, Burera, Rwanda

**Keywords:** Cancer, Communication, Training, Breaking bad news, Doctor-patient relations, Africa, Rwanda

## Abstract

**Background:**

The burden of advanced cancer is rising in Africa. Cancer care involves complex conversations between providers, patients, and families. International guidelines recommend communication skills training for all cancer care providers, and patient-provider communication and training needs are strongly influenced by culture. As oncology and palliative care capacity expand in African settings such as Rwanda, participatory research is needed to culturally adapt communication skills training to best fit local contexts.

**Methods:**

Guided by the Cultural Adaptation Process model, this study aimed to set the stage for adaptation and implementation of serious illness communication skills training in the Rwandan context. We conducted focus group discussions with interdisciplinary cancer care providers at Butaro Hospital in Rwanda to understand their communication training priorities and describe pertinent contextual factors. The focus groups were audio-recorded, transcribed, and analyzed using the framework method of thematic analysis.

**Results:**

A total of 17 cancer care providers participated in one of three focus groups, including six physicians, seven nurses, two psychologists, and two social workers. Participants identified delivering bad news and responding to emotion as the most challenging aspects of clinical communication and the highest priorities for training. They expressed concerns about the psychological toll of difficult conversations on providers, advocating for future trainings to include burnout mitigation strategies. Participants described several key contextual factors that should inform adaptations of communication training for Rwandan cancer care. These include barriers common to low-resource settings as well as several local assets: interdisciplinary collaboration, dedicated clinical psychologists, group counseling sessions, peer support among patients, and strong community networks. Several findings will be directly applied to the design of an initial pilot communication training in Rwanda. Areas requiring further investigation and opportunities to broaden the scope of communication interventions beyond patient-provider encounters were identified.

**Conclusions:**

This study sets the stage for adapted communication skills training in Rwanda that is guided by the priorities and recommendations of local cancer care providers. Several pertinent cultural and structural factors were identified that are common across diverse African settings. Therefore, our training adaptations, as well as the methodology used for adaptation, have the potential for widespread reach.

**Supplementary Information:**

The online version contains supplementary material available at 10.1186/s12904-025-01879-z.

## Introduction

The burden of cancer is rising in Africa, and most patients present with advanced, incurable disease [1]. Cancer care involves complex conversations between providers, patients, and families, including the delivery of bad news, treatment decision-making, and transitions from cancer-directed therapy to end-of-life care. High-quality communication can improve patient well-being, support families, decrease provider burnout, and lower healthcare costs [2–5]. Conversely, poor communication can play a central role in the physical and psychological suffering of patients [6]. 

Studies from diverse African cancer care contexts show that patients are often inadequately informed of their diagnosis, prognosis, and expected treatment outcomes and inadequately included in care decisions [7–14]. Across many settings, providers are ill-prepared to deliver bad news, respond to patients’ emotions, and engage in shared decision-making [14–19], which contributes to provider burnout, a threat to the already strained African oncology workforce [20]. Thus, improving clinical communication offers an immediate opportunity to reduce suffering among patients, improve morale among providers, and enhance the quality of cancer care in Africa.

Extensive research has shown that providers can improve their communication skills with training, and international guidelines recommend mandatory communication skills training for all cancer care providers [21, 22]. Most serious illness communication skills training programs have been developed in Western high-income countries, where patients often have access to multiple cancer treatment options and potentially intensive end-of-life care amidst a strong cultural emphasis on individual autonomy. Reflecting this, communication guidelines in cancer care focus on identifying patients’ individual goals and preferences to inform shared decision-making [23, 24]. However, clinical communication is significantly influenced by cultural and structural factors [25, 26]. For instance, cultural meanings associated with cancer affect disclosure of a cancer diagnosis, norms around talking openly about death influence prognosis communication, and cultural orientations toward communalism versus individualism may guide family involvement in decision-making [26]. Structural factors such as access to effective treatment options and patient-to-provider ratios also significantly affect communication. Thus, communication training priorities in one context may not be readily transferrable to another.

Rather than “exporting” communication interventions to Africa, participatory research is needed to culturally adapt existing interventions for local contexts [27]. To date, however, few studies have developed or adapted serious illness communication training programs in low- and middle-income countries. In a scoping review of cancer and palliative care communication research in Africa, only two out of 58 studies evaluated communication training interventions, though the majority recommended implementing communication skills training for providers [28]. One of these evaluated a role-playing workshop to teach medical students the six-step “SPIKES” protocol for delivering bad news in Botswana [29] and the other evaluated a pilot webinar-based communication training series based on the “COMFORT” communication model in Kenya [30]. In both cases, a training tool from the U.S. was implemented directly without systematic adaptation. As oncology and palliative care capacity expand in Africa, there is a major opportunity to incorporate locally tailored communication interventions into clinical care.

In Rwanda, oncology and palliative care capacity have grown substantially since the opening of the country’s first cancer care facility, the Butaro Cancer Center of Excellence (BCCOE), in 2012. A prior qualitative interview study among patients receiving palliative chemotherapy and providers at BCCOE found that patients were generally well informed of their diagnosis and the intent of treatment, whereas most had not engaged in discussions about prognosis or in treatment decision-making [31]. Yet these were less important to patients than relational aspects of communication, such as a provider’s interpersonal style, and practical aspects related to enabling self-management. Patients cited power hierarchies between patients and providers as a barrier to communication and providers emphasized health system constraints. Both providers and patients advocated for communication skills training for providers, which had been identified as a need in other Rwandan studies [32, 33]. To address this need, we set out to co-create a communication skills training intervention for the Rwandan context through cultural adaptation of existing interventions. The present study aimed to understand the specific communication training priorities of cancer care providers in Rwanda and to describe pertinent contextual factors. This study sets the stage for the adaptation and implementation of serious illness communication skills training in Rwanda.

## Methods

### Study setting

BCCOE is located at Butaro Hospital, a rural district hospital run by the Rwandan Ministry of Health with support from the non-governmental organization Partners In Health/Inshuti Mu Buzima (PIH/IMB) and academic partners in the U.S. BCCOE provides basic cancer care including pathologic diagnosis, surgery, chemotherapy, and supportive care. Core elements of palliative care are available at BCCOE, such as access to morphine, other symptom management, and supportive counseling by oncology-dedicated clinical psychologists. Whereas patients are referred to BCCOE from across Rwanda for cancer diagnosis and treatment, most are referred back to their local district hospitals for end-of-life care. At the time of this study, cancer care at BCCOE was provided by internists, pediatricians, surgeons, general practitioners, and nurses via a task-shifting model [34], as there were not yet oncologists or palliative care specialists permanently on site. In this model, BCCOE providers routinely consult with U.S.-based clinical advisors for clinical recommendations, teaching, and mentorship during weekly virtual tumor board conferences and regular in-person visits.

### Positionality

This study was led by P.U., a researcher at PIH/IMB with a nursing background and expertise in qualitative methods, and R.J.D., an oncologist and researcher from the U.S. who had previously served as a full-time oncology clinician at BCCOE and subsequently as a clinical advisor. The study team included the PIH/IMB Training Coordinator (I.G.), who was also formally trained in Kinyarwanda translation, the Oncology Program Manager (J.B.B.), and the Director of Oncology (C.S.). The study was guided by local mentor V.C.K., who had pioneered research on patient-provider communication in Rwandan primary care settings [32, 35, 36], and by U.S. mentors with expertise in global oncology (K.V.L.), communication-related implementation science (R.L.S.), and communication skills training and related research (J.J.S.).

### Study design

This study is part of a larger project aiming to adapt a communication training intervention from the U.S. for the Rwandan context. We used the Cultural Adaptation Process model, which outlines a procedure for systematic cultural adaptation of evidence-based interventions in three phases: (1) setting the stage; (2) initial adaptation; and (3) adaptation iterations [37]. As part of the first phase, we conducted three focus groups with physicians, nurses, clinical psychologists, and social workers who care for patients with advanced cancer at BCCOE (herein referred to collectively as “providers”). This paper presents the focus group findings related to communication training priorities and contextual factors in Rwanda per COREQ reporting guidelines [38] (see checklist in Supplementary Files). We subsequently applied these data to make a priori adaptations to the U.S. training intervention and iteratively piloted the adapted training with providers in Rwanda (DeBoer, et al., in press). Focus group findings specific to communication skills training methods and tools are reported in that paper.

The Serious Illness Care Program is an evidence-based multicomponent intervention designed to improve patient-clinician communication in the setting of serious illness such as advanced cancer [39]. This program includes training providers in the use of a structured tool called the Serious Illness Conversation Guide (SICG), which outlines the following steps: set up the conversation; assess patients’ understanding and information preferences; share prognosis; explore key topics, such as patients’ values and fears; and close the conversation [40]. This intervention was originally developed in a U.S. academic oncology setting [41]. We selected the SICG for adaptation because it is designed to guide a full conversation from beginning to end and includes a range of skills within one tool. The SICG was translated to Rwanda’s local language, Kinyarwanda, by P.U. and I.G. and a hard copy was given to focus group participants. A video of local actors simulating a clinical encounter using the Kinyarwandan SICG was shown during the focus groups.

### Study participants

Potential participants were purposefully sampled [42] to include all providers who routinely communicated with patients and families at BCCOE. This target sample included all adult and pediatric oncology physicians (*n* = 8), all oncology-dedicated clinical psychologists (*n* = 2) and social workers (*n* = 2), and a subset of oncology nurses (*n* = 8). Potential participants were invited both by email from R.J.D. and by phone or in-person by P.U. All invitees participated except for two physicians and one nurse who were not available (17 out of 20; 85%). Written informed consent was obtained from all participants by P.U.

The first two focus groups were stratified by profession, with physicians in one group (*n* = 5) and nurses, psychologists, and social workers in the second group (*n* = 6), to avoid potential effects of hierarchical dynamics on participants’ responses. The third group was conducted several months later, after the first two groups were analyzed, and included members of all professions (*n* = 6) because there was only one eligible physician remaining.

### Data collection

Focus groups were facilitated by P.U. with assistance from I.G. using a discussion guide that inquired about participants’ experiences with communication in cancer care at BCCOE, previous communication skills training, current training needs and preferences, and feedback on the SICG (Table 1). After the first two focus groups, the discussion guide was revised to focus on unsaturated themes in the third group. Focus group discussions took place in an office conference room at BCCOE and were moderated in Kinyarwanda with some use of English terms. Discussions ranged from 72 to 93 min and were audio recorded, transcribed verbatim in Kinyarwanda, translated into English, and de-identified. Since personal pronouns are genderless in Kinyarwanda, “they/them” was used unless gender was specified. Transcripts were uploaded to MAXQDA (VERBI Software; Berlin, Germany) for data coding and analysis.

**Table 1:** Summary of focus group discussion guide

Section	Topics covered
Introduction	• Welcome, Objectives, Ground Rules, Warm Up
Patient-provider communication at BCCOE	• [Description of “serious illness conversations”] • *What is your experience with these conversations at Butaro?* • *When it comes to these conversations*,* what works well?* • *When it comes to these conversations*,* what are the challenges?*
Communication skills training	• *In your experience*,* how have you learned communication skills?* • [Description/examples of different methods used for communication skills training] • *Are any of these methods familiar to you?* • *If we are going to design communication skills training for Butaro*,* what do we need to understand about the Rwandan cultural context?* • *What communication skills training methods do you think would be MOST effective in Rwanda?* • *What communication skills training methods do you think would be LEAST effective in Rwanda?*
Serious illness conversation guide (SICG)	• [Short video demonstration of doctor using SICG with patient] • *What are your thoughts about using this guide in Rwanda?* • *Are there any changes you suggest to make the guide better fit the Rwandan context?* • *What are your thoughts about using a guide like this one to practice communication skills in small groups (i.e.*,* role play) in Rwanda?*
Conclusion	• *Is there anything else you would like to add related to patient-provider communication or communication skills training in Rwanda?* • Wrap-up

### Data analysis

Textual data were analyzed by P.U. and R.J.D. using the framework method [43], a type of structured thematic analysis that starts deductively with pre-set objectives (i.e., adaptation of communication training) and maps onto an explicit thematic framework. After an initial stage of familiarization with the raw data, they developed an analytic framework and corresponding coding system that combined conceptual categories in the discussion guide with those that emerged inductively during open coding. The resulting framework included codes related to communication training content (i.e., specific skills) and format (i.e., training methods). Contextual factors were categorized as barriers or facilitators at the patient, provider, health system, and sociocultural levels. P.U. and R.J.D. independently coded each transcript and met to review intercoder agreement. Coded segments of data were categorized according to the analytic framework and interpreted by category to elucidate themes. Findings were presented to BCCOE providers during subsequent pilot communication skills trainings for further feedback (DeBoer, et al., in press).

### Reflexivity

Several aspects of the researchers’ positionality shaped this study. First, the study team included PIH/IMB program leaders and longtime clinical advisor R.J.D. with the expressed goal of improving communication skills among providers, including study participants. This occurred within a strong programmatic emphasis on capacity-building and continuous quality improvement at BCCOE [44]. Thus, this study may have been perceived by participants as an internal capacity-building project and opportunity to express opinions to people in positions of power over their working environment and professional development. Second, facilitators P.U. and I.G. and participants were part of the same BCCOE staff community, and all shared a common identity as Rwandan nationals working in the public healthcare system. This foundation of shared understanding appeared to promote trust and candor among participants. Moreover, a majority of participants had also participated in one-on-one interviews with P.U. for a prior qualitative study about communication at BCCOE [31]. Insights from this prior study and firsthand experience with clinical communication as a former nurse likely influenced the probes P.U. asked in the focus groups. Finally, the researchers’ backgrounds as clinicians, program leaders, and implementation scientists may have biased their orientation in favor of clinical and health system aspects of context rather than sociocultural factors, and toward implementation rather than theoretical considerations. The study design and analysis were also shaped by R.J.D.’s positionality as an American oncologist trained in communication skills through U.S. programs, and by the U.S. collaborators’ perceptions of Rwandan context through an outsider’s lens.

### Ethical review

This study was approved by the Partners In Health/Inshuti Mu Buzima (PIH/IMB) Research Committee under an umbrella protocol approved by the Rwandan National Ethics Committee (No. 804/RNEC/2021), and by the University of California, San Francisco Institutional Review Board (#20-30116). Written informed consent was obtained from all participants.

## Results

A total of 17 interdisciplinary providers participated in one of three focus groups (Table 2). They identified delivering bad news and responding to emotion as the top priorities for communication skills training in Rwanda and expressed concerns about the psychological toll of difficult conversations on providers. Participants discussed several contextual factors that influence clinical communication in Rwanda at the patient, provider, health system, and sociocultural levels (Table 3).

**Table 2:** Participant characteristics

Participant characteristics (*N* = 17)	*N*	%
**Position**		
Physicians	6	35%
Nurses	7	41%
Social workers	2	12%
Clinical psychologists	2	12%
**Gender**		
Female	5	29%
Male	12	71%
**Age**		
< 30 years old	1	6%
30 to 40 years old	8	47%
> 40	8	47%
**Degree of studies**		
Bachelor’s degree or below	10	59%
Masters’ degree and above	7	41%
**Working experience**		
< 5 years	8	47%
*≥* 5 years	9	53%

**Table 3:** Key contextual factors that influence clinical communication in Rwanda

Level	Barriers	Facilitators
Patient	• Lower levels of education • Limited understanding of cancer • Alternative health belief systems	• Higher levels of education • Family member accompaniment • Engagement and empowerment
Provider	• Lack of clear delineation of interdisciplinary roles • Inadequate communication skills training	• Interdisciplinary teamwork • Dedicated clinical psychologists • Experience with conversations over time
Health system	• High patient-to-provider ratios • Staffing shortages and turnover • Risk of misinformation among patients • Cancer treatment resource limitations • Non-disclosure or false hope by external referring providers	• Private room for counseling • Peer support fostered by open ward • Group counseling sessions • Cancer treatment resource availability • Information disclosure by external referring providers
Sociocultural context	• Widespread perception of cancer as a death sentence • Poverty and financial toxicity	• Strong family and community networks and financial support • Increasing public awareness of cancer treatability and survivability

### Communication training priorities in Rwanda

#### Delivering bad news and responding to emotion

Participants identified delivering bad news as the foremost challenge in cancer communication and welcomed the opportunity for training in this skill.

*You must teach us how to disclose bad news; how do you initiate the conversation? How do you tell it to the patient*,* how do you tell it to the family? (CP-2C)*

To participants, “breaking bad news” encompasses disclosure of a cancer diagnosis, discussion of incurability, and, especially, informing patients of a transition from curative to palliative goals of care.

*Bad news comes in the moment we have tried the care*,* the patient has respected all appointments*,* they have taken all medicine*,* they finally relax*,* and we realize that the treatment is not helping. In that case*,* we tell them they are going to shift to palliative care. That’s what I consider as bad news. (RN-3 F)*

Some doctors explained that delivering bad news is so difficult they actually avoid it:

*For every patient I receive and break bad news*,* I keep their image in my mind… I think we do not break bad news in the right way*,* because there are some things you try to evade… even unconsciously*,* because they make you very*,* very uncomfortable. (MD-1E)*

*Sometimes I set an appointment with the patient*,* and I unfortunately apologize and cancel at the last minute. Why? Because*,* I may go there to meet them*,* and when I see the patient and their relatives’ mood*,* I feel I am not ready to announce that bad news. I may then postpone it once*,* twice*,* or even three times. (MD-3 C)*

Several participants attributed the difficulty of delivering bad news to the related challenging task of responding to patients’ emotions:

*No sooner did I tell them about their illness than they broke into crying*,* and I had difficulty calming them down. You can imagine how it feels when you are with someone and they cry. (RN-2B)*

Others attributed the difficulty of delivering bad news to the tension between truth telling and maintaining hope:

*It happens that you give promise of recovery to the patient while you can see well that they will pass away soon. (MD-1E)*

Yet, some—a psychologist and social worker—noted that despite the initial difficulty of receiving bad news, patients need to be informed, and with time they accept the information.

*Of course*,* patients get shocked with the news*,* but if you give them time*,* sometimes they end up telling you*,* “At least I feel relieved now that I know all about my diagnosis; it is better than seeking healthcare unaware of what will happen.” (CP-2E)*.

Participants also pointed out significant heterogeneity among patients in receiving bad news, with some patients exhibiting even greater emotional strength than clinicians.

*Some patients will show severe emotions of sadness [or] hopelessness*,* but there are others who appear to not mind about the news. (MD-1 C)*

*There are patients who*,* even though they seem to hear the bad news for the first time*,* look calm and appear to have already accepted their illness*,* and you even feel surprised that they are emotionally stronger than you as the doctor! (MD-1E)*

#### Impact of serious illness communication on providers

Participants of all disciplines emphasized the negative emotional impact that delivering bad news has on providers.

*Cancer is really different from other illnesses. When you are going to initiate a conversation saying… “We will not cure you” or “We are no longer able to cure you*,*” I feel weak emotionally*,* I feel pressed down by that illness. (MD-1 A)*

*In oncology we have patients who come regularly*,* and you have time to build friendship with them. So*,* when a certain patient is going to change from curative to palliative intent*,* you … ask yourself*,* “Why has this happened to my friend? Why are they not responding to treatment?” and you just feel affected. (RN-2D)*

Several participants related their negative emotions to imagining themselves in a patient’s shoes.

*It’s not easy to tell someone they will not recover from their illness despite the treatment we are giving them. When I have to break such news to a patient*,* I myself feel afraid! Because I am also a human being*,* so I try to stand in their shoes and imagine how I would feel. (RN-2B)*

Multiple participants perceived a need to conceal their own emotions for the sake of patients.

*I am filled with emotions even though I try to not show them. I try to conceal them in order to help the patient. (SW-2 A)*

Several participants expressed concern about the cumulative psychological effects of repeated difficult conversations on providers, invoking concepts of “burnout” and “compassion fatigue.”

*This situation leaves us with psychological burden and affects our quality of life. Even though we sometimes consider [communication] as simple*,* it really affects us a lot. (MD-1E)*

*If the clinician breaks bad news [today] and they also tell similar bad news tomorrow*,* progressively they end up being affected emotionally*,* as the saying goes in French*,* “ce que nous rencontrons nous laissent des traces*,*” which means “what we encounter everyday leaves us with traces.” Those stories remain in their mind and [lead to] burnout. (CP-2E)*

They advocated for future communication trainings to include lessons in managing one’s own emotions and in self-care for burnout prevention.

*You should also think about how to help the doctor manage their own emotions in case they also have emotions*,* what do they do then? …this should also be included in the training. (MD-1 C)*

*In the training… [perhaps] you will teach people how to do “self-care.” If someone has burnout after telling one*,* two*,* three patients*,* “You will not recover from your illness*,*” [they] will get discouraged… Will you include a session to help providers to care for themselves? (CP-2 C)*

### Contextual factors influencing clinical communication in Rwanda

Participants described several barriers and facilitators that influence clinical communication and training needs in Rwanda (Table 3).

#### Patient-level factors

Participants asserted that many patients and caregivers at BCCOE have limited understanding of cancer and may prefer not to engage in information exchange or decision-making, instead deferring entirely to the provider.

*Most of our patients have a low level of understanding. When you try to explain to them*,* they block the conversation from going on. They tell you*,* “Doctor*,* you are the one to have studied that. I agree with whatever you are doing.” (RN-3D)*.

Participants suggested that communication trainings be adapted to meet the needs of patients who have low education or follow alternative health belief systems.

*We receive many people whose level of poverty is very high … and their behaviors regarding cancer*,* their perceptions are also different. You will hear someone saying*,* for example*,* “A witch doctor will help me*,*” or “I will go to pray to God.” …Now that you are about to prepare the communication skills training*,* you should … adapt those techniques to the lower level of education of our patients. (MD-1D)*

Participants also noted the importance of family members who can bridge gaps in understanding to facilitate communication.

*Before holding a conversation with a patient about their prognosis*,* we should ask if they have somebody close to them who has a level of understanding a bit higher than the patient’s. … Involving a third-party person who is their close relative or caregiver whom the patient was involved in choosing would help. (RN-3D)*

#### Provider-level factors

Communication was generally seen as a shared responsibility among clinicians, psychologists, and social workers, with interdisciplinary teamwork facilitating high-quality communication.

*If the information was announced by a physician*,* they might not master some aspects*,* such as social or psychological ones. When sharing the information with a patient*,* there should be a doctor*,* social worker*,* and psychologist at least. (MD-3 C)*

Teamwork, and particularly the collaboration of a psychologist, also alleviates the emotional burden experienced by clinicians.

*It’s fortunate that nowadays we have a psychologist… Sometimes I feel positive emotional relief because I know that at some point*,* I will pass the ball onto them. Even though there are some things I have to explain to the patient as a doctor*,* the situation is different when they are helping me. (MD-1 A)*

Yet views were mixed about the extent to which doctors should delegate difficult conversations to others. Participants reported that doctors often stop delivering serious news to patients in response to strong emotions and instead refer those patients to psychologists:

*When [the patients] start crying*,* even if the doctor has said only one word*,* they immediately stop … and say*,* “Where is [psychologist’s name]? Find them.” That is*,* the doctor’s conversation ends there. (RN-3E)*

*It happens that the doctor*,* upon seeing how much the patient is overcome with emotions*,* refrains from telling all the information to the patient. When we [psychologists] come to know [this]*,* we approach the doctor so that they can give us the full information*,* and we can then tell it to the patient in a detailed way. (CP-2E)*

Some clinicians upheld this practice as appropriate or preferable.

*It’s up to the psychologist to go deeper in the details. Honestly*,* we as clinicians*,* we don’t go deep. It’s difficult for clinicians to sit and go through all these steps [in the SICG] … We just tell them their prognosis and refer them to psychologists. (RN-3 A)*

*I often wish that I could receive patients after they have already been told their bad news by someone else*,* not me*,* so that I am not the one to tell them about it. (MD-1E)*

In contrast, others asserted that it is the doctor’s responsibility to discuss difficult news with patients first, referring to the psychologist when additional support is needed.

*Normally*,* even if we wish to have a psychologist*,* counseling should actually be carried out by a doctor. … In general*,* it is the doctor who is expected to announce the diagnosis to the patient and the psychologist can do what is known as “maintenance*,*” or make the patient remain calm. (MD-1D)*

Furthermore, participants noted that a lack of clear delineation of each provider’s role can be a barrier.

*As we are a team and our roles are different*,* we don’t have guidance [to] indicate*,* “when you receive a patient*,* you as a nurse or a physician*,* you must start from here and finish by here; you will say this*,* and the next care provider will say this…” When there is no guidance that makes clear each one’s role*,* this is a barrier. (RN-2 F)*

Also at the provider level, participants indicated that inadequate communication training is a barrier, whereas clinical experience over time is a facilitator.

*Many of us dread breaking bad news because we… did not have any practical training on how to do that. (RN-2B)*

*It is thanks to experience that one gets familiar… by dint of frequently telling patients about their illnesses*,* you get accustomed to breaking the bad news and you become able to reassure them. (MD-1 C)*

#### Health system factors

All focus groups discussed health system barriers, emphasizing that high patient-to-provider ratios and staffing shortages lead to significant time constraints.

*The number of patients we receive—we have very little time to talk with patients. For example*,* the patients we received today in [clinic]*,* you couldn’t make even 5 min for telling the patient about her illness. (MD-1E)*

W*e are a big team*,* though we are not enough. Why? We have one psychologist who may be needed closely in pediatric ward when we also need his support in onco clinic*,* and at the same time he is as well needed in onco adult ward. That is a barrier on the side of staffing. (RN-2 F)*

High turnover of experienced staff, especially among nurses, is another barrier to communication quality.

*We sometimes have competent nurses with experience. Unfortunately*,* after some time*,* they all quit. I don’t know if the new ones are able to take a case in oncology and have a serious conversation while they have got no experience. (RN 3 F)*

While lack of privacy in the open ward can be a barrier, participants indicated that generally there is a separate room available for counseling. Moreover, the close proximity of patients on the ward can facilitate peer-to-peer support.

*Sometimes you tell bad news to a patient and they feel very sad*,* but when another patient next to their bed shares their own experience*,* and how they tried to cope with their illness*,* you can see the patient in different mood next time; they are smiling and have overcome the burden of the problem. Patients help each other. (RN-2D)*

However, some participants pointed out the drawbacks of patient-to-patient communication, including the risk of spreading information that may be true for one patient and not another.

*If we tell patients that they are in the end-of-life stage*,* …normally*,* that communication is between you and the patient*,* right? However*,* they sometimes … immediately unload their problem on [other patients]*,* [who] immediately understand it that way. Later*,* when you talk to somebody else with a different problem*,* you realize they are basing on what they heard from your previous patient. (RN-3 A)*

All focus groups discussed the common practice at BCCOE of group counseling, in which a social worker, nurse, or psychologist provides education and supportive counseling to a group of patients. This was largely seen as a facilitator of both communication efficiency and peer support.

*There is also a strategy of talking about patients’ diagnosis in a group*,* because when patients are together*,* they comfort one another*,* as opposed to when you break the bad news when the patient is alone. (MD-1E)*

However, some participants expressed concern that the information delivered to patients in group counseling may not be adequately individualized.

*I don’t agree with the way … [they] often combine patients with breast cancer*,* prostate [cancer]*,* locally advanced cancer*,* metastatic cancer*,* etc. simply because they are new cases. (RN-3 A)*

Another major theme at the health system level was the influence of resource availability. Participants emphasized that disclosing a cancer diagnosis is much easier when they are able to offer treatment.

*It all depends on the type of cancer that they have; when you … find that their cancer is among those that we treat or those we know are curable*,* that gives you courage to explain to them. (MD-1D)*

Conversely, communication is more difficult when there is no treatment to offer. This is especially true when effective treatment may be available in other settings but is inaccessible at BCCOE.

*If the illness is at advanced stage or you don’t have medications for [it]*,* it is very distressing… because the patient rushes to see you for their rescue only to find that you cannot help them at all. (MD-1D)*

*What can make the patient not understand or accept their illness… for example*,* clinicians tell the patient*,* “We don’t have treatment for your illness” or “For your illness*,* the treatment can only be found abroad*,*” and the patient doesn’t have financial capacity to afford that treatment. (CP-2 C)*

Finally, participants discussed the influence of clinical communication outside of BCCOE. When patients’ diagnoses are disclosed by an external referring provider, subsequent conversations at BCCOE are easier.

*Because of the long way that people travel from other referral hospitals to Butaro*,* patients do have some information and are even told that they might have cancer. This makes the patient seem ready to accept their diagnosis… they start “self-counseling.” (MD-1E)*.

Yet many patients are not adequately informed of their diagnosis by referring providers, requiring a BCCOE provider to start from the beginning.

*It happens that a patient comes to Butaro upon referral by another hospital but reports that they weren’t told anything about their illness. … They may be experiencing “denial*,*” [or] it may be because really*,* they didn’t tell them what illness they have. In this case*,* you start the conversation over again. (MD-1 C)*

A different challenge arises when patients are assured by referring providers that they will receive cancer treatment at BCCOE, but in fact treatment is not available for their cancer type or stage.

*If you return a patient back home after they were referred here*,* the most difficult thing is that they continue to think they have been denied medications… Other providers at the referring hospital informed such a patient that there are medications at Butaro for their illness*,* and they don’t understand why some patients are getting medications for their cancers while they are not. (MD-1D)*

#### Sociocultural factors

Many participants explained that widespread cultural beliefs equating cancer with a death sentence make discussions about cancer especially difficult.

*The word “cancer*,*” in our culture*,* means the “worst thing you can think of in the world; it is the worst illness of all illnesses that exist.” (MD-1 C)*.

*In Rwandan context … people refer to cancer as “a slow death.” Therefore*,* whenever you tell a patient that they have cancer*,* they exclaim*,* “I will absolutely die!” (CP-2 C)*.

In addition, many patients at BCCOE are affected by poverty, and news related to cancer can have catastrophic financial implications, making delivery of that news very challenging.

*Patients reach Butaro after visiting several hospitals*,* and they have even sold everything they possessed*,* so when they come to Butaro they have become extremely poor. Therefore*,* when they receive such bad news… they start thinking about the rest of their life and the future of their family that is going to be left impoverished*,* and all these thoughts make the patient overwhelmed by hopelessness. (SW-2 A)*

On the other hand, strong community networks that provide psychological, spiritual, and financial support to patients and families are an important asset in the Rwandan context.

## Discussion

Participants across disciplines affirmed that communication in cancer care is very challenging and welcomed the introduction of communication skills training at BCCOE. Delivering bad news and responding to patients’ emotions were identified as the highest priorities for training, whereas shared decision-making was lower priority. Participants advocated for training providers in managing their own emotions for burnout prevention. They described several contextual factors pertinent to clinical communication in Rwanda. These include barriers common to low-resource settings, such as cultural understandings of cancer as a death sentence, high patient-to-provider ratios, and limited access to effective treatments and palliative care. They also described several local assets that facilitate communication: interdisciplinary collaboration, dedicated clinical psychologists, group counseling sessions, peer support among patients, and strong community networks. These findings will inform adaptations to communication skills training in Rwanda, addressing an unmet need to co-create cancer communication interventions in diverse African contexts based on local cultural and structural factors and on the priorities and recommendations of training recipients.

Our participants’ overall emphasis on information disclosure from provider to patient is consistent with existing literature from Africa. In a recent scoping review, most studies (60%) of clinical cancer communication in Africa focused on information disclosure, often framed as “breaking bad news,” as in our study [28]. Fewer studies focused on shared decision-making, and none examined skills related to exploring patients’ goals, values, and preferences, which are a key component of the SICG. These skills were also low priority to our participants, who indicated that most patients defer decision-making entirely to doctors. This finding is corroborated by a prior qualitative interview study of oncology patients and providers at BCCOE [31]. While patients in that study desired more information about their treatment plan, most expressed ambivalence about greater involvement in decision-making. And while they valued trust and rapport with providers, none indicated that they wished their providers asked about their preferences, values, or goals. Providers and patients in Rwandan primary care settings have reported similar priorities [32, 35]. Based on this, we decided to focus our initial communication training on sharing information and to defer training in incorporating patients’ goals and values into decision-making. However, despite norms of paternalistic decision-making in Rwanda, some patients do desire greater involvement [31, 35]. Unsurprisingly, studies of patients’ decision-making preferences elsewhere in Africa also demonstrate significant individual-level variation as well as evolution of trends over time [11–13]. Preferences and best practices related to shared decision-making in the Rwandan context should be explored in future studies.

For our participants, “delivering bad news” includes disclosure of a cancer diagnosis, which is often more difficult due to the widespread perception in Rwanda that cancer signifies a death sentence and by the reality that mortality rates are indeed high. Similar cultural meanings associated with cancer are described in other African contexts [7, 17–19]. This difficulty is compounded when there is no treatment available for the patient at BCCOE, especially if they were assured by referring providers that they would receive treatment. Conversely, discussions are easier when referring providers prepare patients to receive a cancer diagnosis and when treatment is available. This highlights an opportunity to collaborate with referring providers, potentially offering them communication training. Additionally, these findings underscore the critical importance of ongoing investment in early detection and treatment delivery to reduce mortality rates and increase awareness of cancer survivability in Rwanda [45]. 

“Delivering bad news” also includes informing someone that their disease is not curable, which can be especially difficult if they were initially treated with curative intent and the disease subsequently recurs. This difficulty is compounded by limited access to high quality palliative care in Rwanda [46], resulting in a common reaction of perceived abandonment and despair among BCCOE patients referred to their local district hospital for “palliative care.” However, the Rwandan Ministry of Health continues to expand community-based palliative care services [45, 46], and the first specialty palliative care team was established at BCCOE in 2024. These advances may reduce the burden of communicating about transitions from anti-cancer therapy to end-of-life care, as providers will be able to offer higher quality palliative care services.

Many participants attributed the difficulty of delivering bad news to patients’ potentially strong emotional reactions. This is a common concern in other African studies, often given as the rationale for withholding information in patients’ best interest [14–16]. We therefore included the skill of responding to emotion as a major objective in our planned communication training. Multiple participants believed that providers should conceal their own emotions, which has also been reported by Rwandan primary care providers [32], yet some Rwandan patients have indicated that they would like providers to express more empathy [35]. To further develop a culturally appropriate tool for responding to emotion, we collected suggestions of empathic statements in Kinyarwanda from local providers in subsequent work (DeBoer, et. al., in press).

Our participants conveyed strong concern about the emotional and psychological toll on providers of repeatedly empathizing with patients in devastating situations, which is a recognized contributor to burnout [47]. To some extent, increasing providers’ comfort with responding to emotions by providing skills training and a forum to share experiences with difficult conversations may mitigate burnout [48]. Participants also recommended including specific training in managing emotions and self-care. A prior qualitative study at BCCOE recommended these and other strategies for resilience, such as mental health services for providers and social activities for team building [33]. Furthermore, addressing systems-level barriers such as staffing shortages is likely to also improve provider well-being [47]. 

Participants recommended incorporating several contextual factors into communication training. These include barriers common to low-resource settings as well as several assets that can facilitate high quality communication. For instance, clinical communication in cancer care is a fundamentally interdisciplinary endeavor at BCCOE, with clinical psychologists taking a central role. While opinions differed on the extent to which doctors should delegate difficult conversations to others, participants largely supported the current system of psychologists taking on most of the counseling, especially given the time constraints of doctors. We therefore decided to first pilot our adapted communication training with clinical psychologists, both to obtain feedback from these local experts and to cultivate future training facilitators. While interdisciplinary collaboration is undoubtedly an asset, participants cited a need for clearer delineation of roles. This need is not unique to Rwanda; an international consensus conference on palliative care research identified a key evidence gap in how to optimize interdisciplinary roles in communication, including in training nonphysicians to conduct specific tasks currently performed primarily by physicians [49]. Future studies in Rwanda should aim to clarify and optimize interdisciplinary roles in cancer communication.

Another asset at BCCOE is an environment conducive to informal social support networks among patients in the open wards and clinic waiting areas. Peer communication is an important potential facilitator of illness understanding and psychological support, although the risk of misinformation may arise. To better assess illness understanding, adapted communication tools could prompt providers to inquire about information patients may have received from other patients. In addition, existing group counseling practices are an asset that can be further leveraged to enhance communication. Though delivery of individualized information is limited in group settings, patients could be more intentionally grouped for receiving similar information, such as a new diagnosis of metastatic cancer or a transition from curative to palliative goals of care. Group counseling also presents an opportunity to empower patients to articulate their questions and to reflect on their values and goals, so they are better prepared for conversations with doctors. Participants indicated that conversations are much easier when patients are well prepared; thus, providers are likely to welcome a shift toward greater patient empowerment.

Participants recommended that communication training incorporate other key sociocultural elements in Rwanda. As in many African contexts, Rwandan culture reflects the Ubuntu philosophy that an individual person is inseparable from their family and community [50]. Strong family and community networks are generally a major asset that can be leveraged in the holistic care of a patient [51]. Providers should make a deliberate effort to include patient-appointed family members in conversations, conceptualizing the unit of care as the family rather than just the individual patient. This is especially salient because cancer care can have significant and sometimes catastrophic financial consequences for patients and families, and understanding a patient’s treatment options and likely outcomes enables them to make appropriate decisions [52]. Families and communities can also have a major role in the propagation versus debunking of myths about cancer treatment, stigma, and reliance on traditional healers or prayer instead of medical care. For instance, in Rwandan palliative care settings, large, inclusive family meetings provide an opportunity to spread education to the broader community [50]. 

In summary, our findings will directly and indirectly inform adaptations to communication skills training in Rwanda. Several findings will be directly applied to the design of an initial pilot training: objectives will focus on sharing information and responding to emotion; training tools will be modified to reflect common contextual factors; and the initial training will be piloted with clinical psychologists. Other findings require further investigation, such as co-creation of a tool for culturally appropriate expressions of empathy, articulation of best practices for shared decision-making in the Rwandan context, and optimal delineation of interdisciplinary roles. Additional findings highlight opportunities to broaden the scope of communication interventions beyond patient-provider encounters, leveraging potential assets such as group counseling sessions, referring providers, and community networks.

This study should be considered in light of its limitations. First, participants were aware of the study team’s plan to implement communication training at BCCOE, which may have biased their receptiveness to training. Second, our findings of the relative importance of different communication skills according to participants were based on their responses to open-ended questions and to the SICG; we did not systematically ask about each potential skill. It is conceivable that, if asked directly, they might have ascribed greater importance to shared decision-making or eliciting patients’ values, for example. Third, our ability to describe sociocultural factors may have been limited by our reliance on informants from within the sociocultural context only, without an ethnographic component. Finally, as a qualitative study with 17 participants that is focused on a unique context, generalizability may be limited. The strength of this approach, however, is that we were able to collect a more in-depth understanding of how to optimize future communication for this context.

## Conclusion

This study sets the stage for adaptation and implementation of serious illness communication skills training in Rwanda. Our approach centers the perspectives of cancer care providers, who are the intended training recipients. Their priorities and recommendations will be applied to co-create a communication intervention that is tailored to the Rwandan context. While certain cultural and structural factors may possibly be unique to Rwanda, many are common across diverse African contexts. Therefore, our training adaptations, as well as the methodology used, have the potential for widespread reach.

## Supplementary Information

Below is the link to the electronic supplementary material.

Supplementary Material 1.

## Data Availability

The data that support the findings of this study may be made available from the authors upon reasonable request and with the permission of the Inshuti Mu Buzima Research Committee (Kigali, Rwanda).

## Abbreviations

CP: Clinical psychologist
MD: Doctor
PIH/IMB: Partners In Health/Inshuti Mu Buzima
RN: Nurse
SICG: Serious Illness Conversation Guide
SW: Social worker
U.S.: United States
