# Correction: Setting the stage for communication skills training in Rwandan cancer care: a qualitative study of local priorities and key contextual factors

**DOI:** 10.1186/s12904-025-01937-6

**Published:** 2025-12-10

**Authors:** Pacifique Uwamahoro, Ignace Girukubonye, Jean Bosco Bigirimana, Cyprien Shyirambere, Katherine Van Loon, Rebecca L. Sudore, Justin J. Sanders, Vincent K. Cubaka, Rebecca J. DeBoer

**Affiliations:** 1 Francois Xavier Bagnoud, Kigali, Rwanda; 2 Cordaid-Rwanda, Kigali, Rwanda; 3 Partners in Health/Inshuti Mu Buzima, Kigali, Rwanda; 4 University of California, San Francisco, San Francisco, CA USA; 5 McGill University, Montreal, Canada; 6 University of Global Health Equity, Burera, Rwanda

**Correction: BMC Palliat Care 24, 240 (2025)**

**https://doi.org/10.1186/s12904-025-01879-z**

Following publication of the original article [1], the author reported that there were errors that should be corrected in the published article. Please see below.

1. On page 2, the following text should be deleted “Query ID="Q2” Text="Please confirm the corresponding Affilaition is correctly identified and amend if necessary.” Resolved="yes”.
2. On page 3, "in the use a structured tool" should be "in the use of a structured tool".

The original article has been updated.
