# Supplementary material for: Setting the stage for communication skills training in Rwandan cancer care: a qualitative study of local priorities and key contextual factors
Source: BMC Palliat Care. 2025 Sep 30;24:240. doi: 10.1186/s12904-025-01879-z (PMC12487494; doi:10.1186/s12904-025-01879-z)
Supplement: Supplementary file 1 — Supplementary Material 1. [file 12904_2025_1879_MOESM1_ESM.docx]

**Consolidated criteria for reporting qualitative studies (COREQ): 32-item checklist**

| **No. Item** | **Guide questions/description** | **Reported on Page #** |
| --- | --- | --- |
| **Domain 1: Research team and reﬂexivity** |  |  |
| *Personal Characteristics* |  |  |
| 1. Interviewer/facilitator | Which author/s conducted the interview or focus group? | Page 8 (Methods, Data Collection, 1st paragraph) |
| 2. Credentials | What were the researcher’s credentials? E.g. PhD, MD | Credentials are listed here:  Pacifique Uwamahoro, MPH, RN  Ignace Girukubonye, BA  Jean Bosco Bigirimana, BSN, RN  Cyprien Shyirambere, MD  Katherine Van Loon, MD, MPH  Rebecca L. Sudore, MD  Justin J. Sanders, MD, MSc  Vincent K. Cubaka, MD, PhD  Rebecca J. DeBoer, MD, MA |
| 3. Occupation | What was their occupation at the time of the study? | Page 6 (Methods, Positionality) |
| 4. Gender | Was the researcher male or female? | P.U., K.V.L., R.L.S., and R.J.D. are female.  I.G., J.B.B., C.S., J.J.S., and V.K.C. are male. |
| 5. Experience and training | What experience or training did the researcher have? | Page 6 (Methods, Positionality) |
| *Relationship with participants* |  |  |
| 6. Relationship established | Was a relationship established prior to study commencement? | Page 9 (Methods, Reflexivity) |
| 7. Participant knowledge of the interviewer | What did the participants know about the researcher? e.g. personal goals, reasons for doing the research | Page 9 (Methods, Reflexivity) |
| 8. Interviewer characteristics | What characteristics were reported about the inter viewer/facilitator? e.g. Bias, assumptions, reasons and interests in the research topic | Page 9 (Methods, Reflexivity) |
| **Domain 2: study design** |  |  |
| *Theoretical framework* |  |  |
| 9. Methodological orientation and Theory | What methodological orientation was stated to underpin the study? e.g. grounded theory, discourse analysis, ethnography, phenomenology, content analysis | Page 7 (Methods, Study Design) and Page 9 (Methods, Data Analysis) |
| *Participant selection* |  |  |
| 10. Sampling | How were participants selected? e.g. purposive, convenience, consecutive, snowball | Page 8 (Methods, Study Participants, 1^st^ paragraph) |
| 11. Method of approach | How were participants approached? e.g. face-to-face, telephone, mail, email | Page 8 (Methods, Study Participants, 1^st^ paragraph) |
| 12. Sample size | How many participants were in the study? | Page 8 (Methods, Study Participants, 1^st^ paragraph) |
| 13. Non-participation | How many people refused to participate or dropped out? Reasons? | Page 8 (Methods, Study Participants, 1^st^ paragraph) |
| *Setting* |  |  |
| 14. Setting of data collection | Where was the data collected? e.g. home, clinic, workplace | Page 8 (Methods, Data Collection) |
| 15. Presence of non-participants | Was anyone else present besides the participants and researchers? | No one else was present besides the participants and researchers. |
| 16. Description of sample | What are the important characteristics of the sample? e.g. demographic data, date | Table 2 |
| *Data collection* |  |  |
| 17. Interview guide | Were questions, prompts, guides provided by the authors? Was it pilot tested? | Table 1; the guide was not pilot tested. |
| 18. Repeat interviews | Were repeat interviews carried out? If yes, how many? | No, repeat interviews (focus groups) were not carried out. |
| 19. Audio/visual recording | Did the research use audio or visual recording to collect the data? | Page 8 (Methods, Data Collection) |
| 20. Field notes | Were ﬁeld notes made during and/or after the inter view or focus group? | No, field notes were not made during or after the focus groups. |
| 21. Duration | What was the duration of the interviews or focus group? | Page 8 (Methods, Data Collection) |
| 22. Data saturation | Was data saturation discussed? | Page 8 (Methods, Data Collection) |
| 23. Transcripts returned | Were transcripts returned to participants for comment and/or correction? | No, transcripts were not returned to participants for comment and/or correction. |
| **Domain 3: analysis and ﬁndings** |  |  |
| *Data analysis* |  |  |
| 24. Number of data coders | How many data coders coded the data? | Page 9 (Methods, Data Analysis) |
| 25. Description of the coding tree | Did authors provide a description of the coding tree? | Page 9 (Methods, Data Analysis) |
| 26. Derivation of themes | Were themes identiﬁed in advance or derived from the data? | Page 9 (Methods, Data Analysis) |
| 27. Software | What software, if applicable, was used to manage the data? | Page 9 (Methods, Data Analysis) |
| 28. Participant checking | Did participants provide feedback on the ﬁndings? | Page 9 (Methods, Data Analysis) |
| *Reporting* |  |  |
| 29. Quotations presented | Were participant quotations presented to illustrate the themes/ﬁndings? Was each quotation identiﬁed? e.g. participant number | Pages 10-22 (Results section) |
| 30. Data and ﬁndings consistent | Was there consistency between the data presented and the ﬁndings? | Pages 10-22 (Results section) |
| 31. Clarity of major themes | Were major themes clearly presented in the ﬁndings? | Page 10 (Results section; first paragraph) |
| 32. Clarity of minor themes | Is there a description of diverse cases or discussion of minor themes? | Pages 10-22 (Results section) |
